# 

**DOI:** 10.3325/cmj.2011.52.657a

**Published:** 2011-10

**Authors:** Farrokh Habibzadeh, Karen Shashok

**Affiliations:** Medical Education and Research Center, NIOC Health Organization, Shiraz, Iran

**In Reply:** The comments of Dr Wiwanitkit are well taken. In our article (1), our main concern was to emphasize the big difference between plagiarism of ideas and plagiarism of text, especially in scientific writing, where we believe text is just a transfer medium for ideas, which are the essence of scientific work (2-5). Paraphrasing of a scientific text is not always easy, if possible at all (6). Moreover, it has been shown that even well-educated native English speakers have difficulties with text paraphrasing, particularly if the text happens to be a little bit complex (6).

The evolution of the scientific enterprise is strongly dependent upon adding small pieces of knowledge to previous ideas – a process that is not limited to medicine. We feel that eventually science communication may reach the state in which readers of an article will pay attention only to the ideas presented, not the words or phrases used in the paper. Scientists will be recognized for their ideas rather than their words and eloquence (7). This is very similar to computer languages, which consist of only a few keywords. In a computer program, the algorithm (idea) used is much more important than the syntax. Based on this view, we believe that soon we will need to develop a better definition of “plagiarism.”

## 

**References**
